# Evaluating the Efficiency and Productivity of Opioid Substitution Treatment Units in Greece: A DEA-Malmquist Analysis

**DOI:** 10.3390/healthcare13080943

**Published:** 2025-04-19

**Authors:** Anastasios Trakakis, Athanasios Theocharis, Panagiotis Prezerakos

**Affiliations:** 1 Department of Nursing, Faculty of Health Sciences, University of Peloponnese, 22100 Tripoli, Greece; 2 National Organization for Prevention and Addiction Treatment (NOPAT), 10433 Athens, Greece

**Keywords:** efficiency, productivity, data envelopment analysis, Malmquist productivity index, addiction treatment

## Abstract

Background: This study examined shifts in the productivity and efficiency of Opioid Substitution Treatment (OST) units in Greece from 2019 to 2022. OST units address withdrawal symptoms in individuals discontinuing psychoactive substances. They also offer mental health care, primary healthcare, psychosocial support, and other integrated services, aiming to provide holistic addiction treatment and promote social reintegration. Methods: We assessed the contributions of 54 OST units addressing opioid addiction using non-parametric Malmquist Data Envelopment Analysis (DEA). Data were collected from all OST units operating in Greece during this period, with a focus on key external factors such as the COVID-19 pandemic and rising global trends in stimulant and synthetic opioid use. Results: The analysis revealed a productivity decline in 2020, followed by improvements in the next two years. However, technical efficiency declined, suggesting a decrease in resource utilization. Conclusions: This dip in efficiency likely reflects the impact of emerging drug trends, particularly stimulants and synthetic opioids, which lack standardized treatment protocols. These findings highlight the urgent need for new treatment options to address evolving addiction trends. The study also underscored the need for improved data collection and monitoring to optimize resource allocation and enhance operational efficiency in OST units. Strengthening evidence-based policies and expanding access with low-threshold treatment services could improve patient outcomes and the overall effectiveness of OST programs.

## 1. Introduction

This study evaluated the efficiency and productivity of Opioid Substitution Treatment (OST) units operated by the Organization Against Drugs (OKANA) in Greece from 2019 to 2022. These units provide substitution therapy with buprenorphine or methadone, along with mental health support, primary healthcare, and psychosocial services to facilitate addiction treatment and social reintegration [1]. By analyzing changes in technical efficiency and productivity over this period, the study aimed to identify key strengths and areas for improvement, optimize resource allocation, and enhance the effectiveness in operations of OST units within the public health framework. Additionally, this paper seeks to provide insights into the strengths and weaknesses of OST units, potentially laying the groundwork for further research in the field of addiction treatment and policy development.

OKANA is the largest government agency in Greece with the aim of preventing and addressing addiction, addictive behaviors, and addiction-related issues in the country. Within its scope of responsibilities, OKANA covers areas such as prevention, harm reduction, treatment, and the social reintegration of individuals facing addiction issues, whether related to legal or illegal substances [1].

In Greece, the total number of individuals using opioid drugs ranges from 1.5 to 2.3 per 1000 persons. This provides us with an estimate of 12,351 active opioid drug users in the country (95% confidence interval: 9920–15,746), as reported in the European Monitor Center for Drugs and Drug Addictions (EMCDDA) Drug Report, 2022 [2].

Cocaine and other stimulants, such as crystal methamphetamine, are gaining popularity in Greece, following the trend seen in other European Union countries. However, heroin remains the primary substance of choice among individuals using drugs, accounting for 53% of the population [2]. Additionally, the EMCDDA’s European Syringe Collection and Analysis Project Enterprise, widely known as the ESCAPE project, which focuses on the collection and analysis of syringes containing blood residuals found in the streets, revealed that the majority of these syringes in Greece contained a combination of substances. For instance, in Thessaloniki, 50% of the syringes contained both heroin and cocaine, commonly referred to as a “speedball” in street language [3].

In 2017, a total of 2593 individuals sought and eventually entered treatment for their addiction. The annual numbers remained relatively stable in the next years, except during the COVID-19 pandemic in 2020, when there was a 17% reduction in the number of people seeking treatment [2,4].

In 2017, approximately 9388 people were receiving Opioid Substitution Treatment (OST). Today, however, the number has decreased to 7340 individuals. This decline reflects a diminishing trend in heroin use within the country. Treatment for individuals with opioid addiction in Greece includes programs that provide substitution therapy alongside mental, psychological, and social support, as well as “drug-free” programs where individuals receive intensive mental, psychological, and social support without the use of substitution therapy [1].

Currently, there are 58 OST units of OKANA, covering the entire spectrum of needs for individuals looking to overcome their addiction. Treatment includes the administration of buprenorphine or methadone to address withdrawal symptoms that individuals often experience when discontinuing the use of psychoactive substances [5]. Specifically, OST units administer Prenorvine tablets and liquid methadone (10 mg/mL), manufactured by the Institute of Pharmaceutical Research & Technology (IFET) in Greece. Moreover, during COVID-19, take-home therapy was provided, including Libroxar, which contains Buprenorphine and Naloxone [1]. Additionally, mental health services, primary healthcare services, psychosocial support, and other accompanying services are provided to achieve holistic addiction treatment and social reintegration [1].

OKANA’s OST units are located within the country’s hospitals and cover most of the territory. Among the 58 OST units, 24 are located in Attica, 11 in Thessaloniki, and 23 in various regions of Greece. Two OST units also operate within the prisons of Korydallos in Athens and Agios Stefanos in Patra [1]. These units were excluded from the study as they operate in a different context, targeting a specific category of individuals, and their operational methods cannot be easily compared to the other units operating within hospitals. Moreover, four out of fifty-eight OST units were excluded from the analysis due to a lack of data to avoid bias.

The contribution of OST units to address addiction has been emphasized internationally [6,7], with significant focus at both the European and global levels on evaluating their effectiveness concerning resource allocation [2,8,9]. However, in both Greece and abroad, the efficiency and productivity of OST units have not received comparable attention, creating a significant research gap. While previous studies have examined treatment approaches and economic aspects [10,11,12], they have often overlooked other critical factors such as mental health consultations, primary care services, and operational elements like staffing levels and associated costs. This study conducted an operational evaluation, considering the impact of the pandemic alongside other external and internal factors, to inform policymaking and enhance decision-making beyond treatment efficacy.

## 2. Materials and Methods

### 2.1. Data Envelopment Analysis and Malmquist Index

Efficiency measurement in research often relies on econometric models, hinging on certain assumptions, mathematical functions, and techniques. These models typically fall into the category of parametric methods for estimating efficiency. However, the healthcare sector presents unique challenges for parametric methods due to the intricate and less definable nature of healthcare processes [13,14]. Notably, parametric methods tend to perform better when dealing with either single inputs or single outputs and necessitate assumptions regarding the model type. In this paper, our analysis encompasses multiple inputs and outputs, a scenario that poses challenges for parametric methods, particularly in model specification. To overcome these challenges, the non-parametric method of Data Envelopment Analysis (DEA) is used, which is also commonly favored in healthcare research for its versatility and adaptability [15].

DEA, developed by Charnes, Cooper, and Rhodes in 1978 [16], represents a significant advancement in efficiency analysis, building upon earlier work by Farrell in 1957 [17], which was rooted in the concepts of Debreu (1951) and Koopmans (1951) [18,19,20]. DEA is essentially a linear programming approach aimed at estimating relative efficiency among production units, often referred to as Decision-Making Units (DMUs), that employ multiple inputs and outputs [15]. Each production unit is efficiency is assessed individually, making them directly comparable, as they share identical functions, resource consumption (inputs), and product or service generation (outputs), differing solely in price levels. In this context, inputs denote the resources utilized in output production while outputs signify the products or services generated by DMUs [15].

The advantages of DEA are multifaceted. Firstly, it is firmly rooted in robust economic theories and methods. Secondly, DEA centers its analysis on relative efficiency rather than absolute efficiency, providing valuable insights into unit performance comparisons. Furthermore, DEA’s capability to simultaneously incorporate numerous inputs and outputs into the model in question, regardless of differing units of measurement, is a notable asset. Additionally, it operates without the need for prior knowledge of specific input–output functions, a particularly advantageous feature in the complex healthcare domain. Nonetheless, it is essential to acknowledge DEA’s limitations. Challenges arise in the selection of appropriate inputs and outputs and in defining the model with reliable data. The method can also be sensitive to outliers, potentially affecting results [21]. DEA was chosen over Stochastic Frontier Analysis (SFA) because it is a non-parametric approach that does not require a predefined functional form, making it more flexible in evaluating OST unit efficiency. Moreover, “DEA is particularly suitable for healthcare settings where inputs and outputs are diverse and difficult to model with a specific functional relationship, unlike SFA, which assumes a predefined production function and separates inefficiency from random noise” [22].

To address these limitations, this study employed “clean data”, ensuring that the dataset was free from inconsistencies, errors, or missing values in input and output variables, thereby enhancing its reliability for analysis. Moreover, this study adhered to a practical rule, ensuring a satisfactory level of DEA’s discriminatory power. The practical rule stipulates “that the number of DMUs should be greater than or equal to three times the sum of inputs and outputs (*n* ≥ 3 × (m + s)) to ensure reliable results” [23].

Efficiency estimation within DEA consists of two stages: first, the calculation of the frontier of best performance, comprising efficient DMUs (100% technical efficiency); and second, the computation of radial distances from the remaining, less efficient DMUs to this frontier [13,24].

DEA measures technical efficiency with two orientations: “The input orientation binds outputs to a linear programming equation that minimizes inputs, while the output orientation binds inputs to maximize outputs” [16,21]. Also, DEA can be employed with two methods: constant returns to scale (CRS) or variable returns to scale (VRS). CRS assumes all DMUs are operating optimally while VRS considers variations in efficiency levels [16,21].

A disadvantage of DEA is that it cannot analyze the impact of time on efficiencies. To address this, the Malmquist productivity index (MPI) is utilized alongside DEA, allowing for the assessment of productivity and efficiency changes over time [25], which was the purpose of this study.

MPI DEA is a non-parametric mathematical programming approach “commonly used to incorporate panel data into analyses and calculate indices for total factor productivity, technical efficiency, pure technical efficiency, scale efficiency, and technological change over time” [26].

In this paper, the input-oriented MPI DEA is employed due to its suitability for the healthcare sector, where controlling inputs is often more feasible than directly influencing outputs, and the availability of panel data. Input-oriented MPI DEA is widely applied in healthcare settings, where resource regulation plays a crucial role in optimizing performance. This approach was also particularly relevant to our study since it provides results that can guide management decisions on resource allocation and utilization. By leveraging these insights, OST units can take targeted actions to enhance efficiency and productivity, ensuring optimal resource use in the future [27,28,29].

While parametric and non-parametric methods have been applied extensively in assessing hospital and healthcare service efficiency [30,31], this study focused specifically on addiction treatment within the domain of public health. While research on the efficiency of OST units in Greece is limited, this study represented one of the first to apply MPI DEA to assess their productivity and efficiency over time.

### 2.2. The Model: Input-Oriented MPI DEA

The primary objective of this research was to assess the technical efficiency and productivity of the 54 OST units operated by OKANA in Greece during the period from 2019 to 2022. While this paper provides an overview of DEA and MPI, it is essential to acknowledge that a comprehensive mathematical analysis of these methodologies exists within the available literature.

Before delving into the MPI, we will first analyze DEA. The mathematical analysis pertains to an input-oriented DEA model under the assumptions of CRS and VRS as both assumptions are crucial for estimating MPI [26].

The analysis operates under the assumption that there are N Decision-Making Units under examination, each employing K inputs to generate M outputs. This leads to the construction of two matrices: a KN matrix representing the utilized inputs (referred to as X) and an MN matrix representing the generated outputs (referred to as Y) [24]. To assess the efficiency of each DMU, T.J. Coelli introduced a mathematical linear programming equation in 1996, which calculates “the ratio of all outputs over all inputs:

   minimize θ, λ θ, subject to:           −yi + Yλ ≥ 0; θxi − Xλ ≥ 0; λ ≥ 0.

Within this equation, θ serves as a scalar taking values within the closed interval [0, 1] and serving as an indicator of the efficiency level of the DMUs, and λ represents an N × 1 vector of constants. DMUs achieving a θ value of 1 operate at optimal efficiency, whereas values less than 1 signify inefficiency” [24]. It is worth noting that this mathematical problem necessitates solving the equation N times for each DMU [24].

The linear programming problem described above “operates under the CRS assumption, which implies that all DMUs operate at an optimal scale. In contrast, the VRS model avoids this assumption, considering potential scale efficiencies” [26]. When adopting the VRS assumption, an additional constraint, N1′λ = 1, is incorporated into the CRS linear programming problem. This extra constraint is introduced to quantify scale efficiency effects, thus partitioning technical efficiency into pure technical efficiency and scale efficiency for each DMU [24,26].

The MPI represents an extension of DEA tailored to measure productivity changes over time for each DMU while dissecting these changes into those stemming from alterations in technical efficiency and those attributed to advancements or declines in technology levels [24]. MPI DEA is ideally suited for panel data, eliminating the need to select between the CRS or VRS approach since they yield equivalent results. In MPI DEA estimation, both the CRS and VRS approaches are employed to compute the diverse distances forming the foundation of Malmquist indices [26,32].

The MPI DEA method offers two orientations: input and output orientations. In the input orientation, production is characterized by calculating the minimal proportional decrease in the input vector, considering the output vector. Conversely, in the output orientation, production is defined by computing the maximal proportion increase in the output vector, given the input vector [33].

In this study, panel data spanning four years (2019–2020–2021–2022) were examined, forming three distinct periods, comparing years from t to t + 1 (2019–2020, 2020–2021, and 2021–2022). The computation of the MPI entailed estimating the distances for each of these three periods. A DEA analysis, incorporating both the CRS and VRS assumptions, was carried out for each of the four years (2019, 2020, 2021, and 2022) to evaluate the efficiencies of the DMUs to estimate the Malmquist indices for the three periods examined. It is important to note that while the efficiencies of the DMUs for the year 2019 were computed, Malmquist indices could not be estimated due to the unavailability of data for 2018, and thus no comparison between 2018 to 2019 could be made.

The Malmquist index, introduced initially in 1970 using Shephard’s distance function, has found extensive applications in various domains where the assessment of efficiency with panel data is required. Färe introduced an output-oriented MPI in 1994 [32]. However, in this paper, we employ the input-oriented MPI approach [24,32,34].

The MPI for a specific period (t to t + 1) signifies the productivity at the production point (xt + 1, yt + 1) relative to the production point (xt, yt) for each DMU, as defined by the following formula [26]:

M (yt + 1, xt + 1, yt, xt) = {[dt + 1 (xt + 1, yt + 1)/dt (xt, yt)] × [(dt (xt, yt)/dt + 1 (xt, yt)) × (dt (xt + 1, yt + 1)/dt + 1 (xt + 1, yt + 1))]1/2}

The MPI equation consists of two fractions, each with distinct interpretations. The first fraction represents the change in technical efficiency over the period (t to t + 1) whereas the second fraction captures the change in technology during the same period [24,26,32,34]. Furthermore, the change in technical efficiency can be dissected into components related to pure technical efficiency and scale inefficiency. Notably, a total productivity growth exceeding one indicates positive productivity growth from time t to time t + 1 [35].

To calculate the MPI equation, the four aforementioned distances must be computed using linear programming methods, which are expressed as follows [26]:

[dt (xt, yt)]^−1^ = minθ,λ θ, subject to: −yit + Ytλ ≥ 0, θxit − Xtλ ≥ 0, λ ≥ 0             [dt + 1 (xt + 1, yt + 1)]^−1^ = minθ,λ θ, subject to: −yi,t + 1 + Yt + 1λ ≥ 0, θxi,t + 1 − Xt + 1λ ≥ 0, λ ≥ 0          [dt (xt + 1, yt + 1)]^−1^ = minθ,λ θ, subject to: −yi,t + 1 + Ytλ ≥ 0, θxi,t + 1 − Xtλ ≥ 0, λ ≥ 0       [dt + 1 (xt, yt)]^−1^ = minθ,λ θ, subject to: −yi,t + Yt + 1λ ≥ 0, θxi,t − Xt + 1λ ≥ 0, λ ≥ 0

It is worth noting that this paper involves the calculation of N(3T−2) linear programming equations [33,36].

### 2.3. Data

The dataset employed in this study, encompassing both inputs and outputs, spans the years from 2019 to 2022. Out of the 58 OST units under the operation of OKANA across Greece, this study’s focus was centered on 54 of these units, with four units being excluded due to constraints related to data availability and quality. This exclusion was to prevent arbitrary estimations and reduce the potential for bias within the analysis. The selected 54 healthcare facilities represented a significant majority, accounting for 93.10% of the total units. Furthermore, these units were distributed across various regions in Greece. This comprehensive approach was intended to bolster the robustness of the evaluation process [37,38].

This study adhered to established criteria governing the execution of MPI DEA, thereby safeguarding the comparability and validity of efficiency measurements for OST units. Specifically, it is important to highlight that all 54 OST units within this analysis employed identical categories of inputs and generated identical categories of outputs. Variability only existed in terms of the quantities utilized. Additionally, each input and output category was included for all 54 Decision-Making Units (DMUs). Within this sample, every DMU effectively utilized at least one input while simultaneously producing at least one output [34,39].

The analytical framework incorporated three distinct outputs to assess the domains of technical efficiency, technology, and overall productivity for each OST unit. Collectively, these outputs spanned the comprehensive spectrum of services delivered by each OST unit. In addition, the analysis incorporated four input variables, which reflected the aggregate staffing levels and resources requisite for the operational functions of OST units. These inputs were vital in facilitating patient therapy, psychosocial support, and various other essential healthcare services.

The dataset provided below encompasses measurements of all 54 OST units, documented on an annual basis to facilitate the subsequent MPI DEA analysis. The variables considered for both inputs and outputs are as follows:
Outputs:Number of Patients receiving therapy, psychosocial support, and healthcare services—output 1Total Number of exams conducted for parallel drug use—output 2Total Number of psychosocial support sessions—output 3Inputs:Total number of staff occupied—input 1Total expenses for payroll—input 2Total expenses to cover the operational costs of the OST unit—input 3Total expenses for medication costs (methadone and/or buprenorphine) necessary for providing therapy—input 4

It is important to emphasize that all inputs and outputs considered in this paper were measured for the years spanning from 2019 to 2022. The summary statistics presented in Table 1 encompass key metrics such as the minimum, maximum, mean, and standard deviation for each input and output variable incorporated in the analysis.

## 3. Results

The measurement of productivity and efficiency for each of the 54 OST treatment units was conducted through input-oriented MPI DEA using DEAP ver2.1 [26].

This analysis evaluated the performance of 54 OST units in Greece across three periods: 2019–2020, 2020–2021, and 2021–2022, using MPI DEA methodology, comparing each year with the next. Key metrics analyzed included efficiency change (Effch), technological change (Techch), pure technical efficiency change (Pech), scale efficiency change (Sech), and total factor productivity change (Tfpch) [17]. Each metric provided insights into shifts in OST unit productivity change from one year to another.

For each period, indicators are summarized in Table 2, with indices over 1 indicating progress and values below 1 indicating declines, while a value of 1 reflects no change comparing one year to the next. The table details the number of DMUs that experienced increases, declines, or stability in productivity across the studied periods. Moreover, overall results for all three periods have been compiled to present a comprehensive view of OST unit performance during these years, which is critical for understanding the change in technical efficiency and technology, as well as for identifying potential areas for improvement in addiction treatment services.

In the first period (2019–2020), only four OST units demonstrated an improvement in total productivity, while fifty units experienced a decline. During the second period (2020–2021), 39 units showed productivity gains whereas 15 units saw declines. In the third period (2021–2022), 36 OST units saw productivity growth, with 18 units reporting reduced productivity. When assessing productivity across all three periods combined, 11 DMUs achieved an overall increase in productivity while 43 DMUs faced an overall decline.

In Table 3, the OST units that progressed, regressed, or remained constant in terms of technical efficiency change, technological change, and productivity change were examined.

As shown in Table 3, OST units displayed fluctuating trends in technical efficiency, technological change, and overall productivity across the three periods. The analysis indicated that productivity declined significantly in the initial period (2019–2020) but showed improvement in the subsequent years (2020–2021 and 2021–2022). To assess these changes, the difference between technical efficiency change and technological change was calculated for each unit. Positive values for this difference suggested that productivity changes were primarily driven by technical efficiency improvements while negative values indicated that changes were primarily due to technological shifts for the DMU [40,41].

Between 2019 and 2022, productivity shifts among OST treatment units were attributed mainly to technological changes for 23 units while 31 units experienced changes primarily due to variations in technical efficiency.

Productivity drivers varied by period. In the first period (comparison between 2019 and 2020), productivity levels declined due to technological changes that affected the production frontier, resulting in generally low productivity among DMUs. In contrast, during the second and third periods, technology expanded the production frontier, boosting overall productivity despite reduced technical efficiency in many OST units. These gains suggest that technology, rather than efficiency, was the primary driver of productivity in these years.

Overall, technology demonstrated considerable volatility across the observed periods, significantly impacting OST units’ productivity. Units that displayed high volatility in productivity also showed marked technological fluctuations, underscoring that productivity changes were largely driven by shifts in technology. During this period, the COVID-19 pandemic likely contributed to the initial decline in productivity observed in the first year of the study. However, the introduction of take-home therapy helped mitigate this trend by expanding the production frontier, leading to improved OST unit productivity in the following years. Similarly, the introduction of generic buprenorphine further expanded the production frontier, enhancing both the technological capabilities and productivity of OST units. Regarding efficiency, the decline in the number of OST unit clients was likely influenced by the rising prevalence of synthetic opioids and stimulants, a global trend that underscores the urgent need to develop and integrate OST treatments for these substances. Adapting OST units to meet this emerging challenge is crucial to ensuring comprehensive care for affected populations [2,3,4,8].

## 4. Discussion

The 2019–2022 period was chosen to capture major external influences on OST treatment units, such as the COVID-19 pandemic in 2020, along with the emerging global trend of stimulant and synthetic opioid use [2,8,9]. These factors were successfully incorporated into the study, with the technology decline observed among DMUs comparing 2019 with 2020 likely influenced by the pandemic-related lockdowns in Greece, along with other challenges affecting OST units. In the subsequent periods, productivity and technological capabilities improved as OST units adapted to new challenges by offering take-home therapy during the lockdowns for individuals facing addiction [1].

Following the pandemic, take-home therapy continued to be an option for patients in OST programs. Research by O’Connor et al. aimed to identify factors that contribute to patient retention in OST and the risks that lead to treatment dropout [42]. Their findings did not establish take-home therapy as a key determinant of retention. However, they emphasized that many OST patients experience isolation, a lack of social support, and restricted access to technology, all of which can hinder treatment adherence and outcomes. Additionally, individuals struggling with mental health issues reported that the isolation caused by lockdowns further deteriorated their well-being [42]. Similar difficulties were observed in Scotland and other parts of the UK, particularly in rural areas where transportation challenges created additional barriers to accessing treatment [43]. A separate study by Adams et al. [44] analyzed the effects of loosening restrictions on take-home doses during COVID-19. This systematic review incorporated 40 studies, primarily from North America and the UK. The quantitative data hinted at a possible correlation between take-home doses and improved retention, though no link was found between take-home doses and increased illicit substance use or overdose risk. The qualitative analysis revealed that take-home doses helped minimize patients’ exposure to unregulated substances and stigma while reducing conflicts between work and treatment. While some individuals faced difficulties in managing their medication, the majority reported reduced anxiety, increased autonomy, and a stronger sense of control over their lives [44]. Overall, keeping take-home therapy available could offer significant benefits to patients, but additional supportive strategies should be explored. Enhancing long-term treatment outcomes may require integrating take-home therapy with alternative service delivery models [44], such as telehealth consultations and virtual support systems, to provide more comprehensive care.

In 2021, the adoption of generic medications (generic buprenorphine) further reduced treatment costs per person [1], contributing to technological and productivity advancements, as well as expanding the efficiency frontier and overall treatment capabilities. However, the technical efficiency of DMUs was still limited, possibly due to a reduced number of patients who were shifting to new substances (stimulants and synthetic opioids), reflecting decreased resource utilization. Although productivity levels improved in 2021 and 2022, some OST units have yet to reach their pre-2020 efficiency and productivity levels.

The findings of this study underscore the urgent need for evidence-based policy interventions to improve the efficiency and accessibility of opioid substitution therapy (OST) units. The correlation between substitution therapy and opioid-related adverse events further reinforces this imperative [45,46,47,48]. Additionally, the increasing prevalence of synthetic opioids and stimulants presents new and complex challenges, which have become critical and require urgent attention more than ever before [2,3,4,8]. Globally, opioid addiction treatment has increasingly relied on medications such as methadone, buprenorphine, and buprenorphine/naloxone, which have demonstrated effectiveness in reducing opioid-related harm [49]. However, to optimize treatment outcomes, policy frameworks must prioritize the allocation of resources to ensure that patients receive comprehensive care that addresses not only their addiction but also their broader needs such as mental health care, housing, and employment support [50].

Improving the operational efficiency of OST units requires more effective resource allocation guided by evidence-based decision-making. Policymakers should invest in robust monitoring and evaluation systems to track key performance indicators, including patient retention, treatment adherence, and the impact of external factors such as the rise of synthetic opioids and stimulants. Such data can highlight service gaps and inform targeted improvements. In addition, reducing the stigma associated with OST programs is essential as stigma remains a major barrier to patient engagement and retention. Public awareness campaigns and training for healthcare professionals can help create a more supportive treatment environment, ultimately improving patient outcomes [51].

A significant challenge in the global context is the increasing prevalence of synthetic opioids and stimulants, which have introduced new complexities to addiction treatment. Research indicates that the use of these substances is rising, and global health organizations, such as the EMCDDA, WHO, and United Nations, have called for the development of targeted treatment options for these drugs [2,3,4,8]. Given the rapid rise of these drug trends, there is an urgent need to move beyond harm-reduction strategies and develop effective treatment options [2,8]. The introduction of new treatment protocols for synthetic opioids and stimulants is essential to keep OST programs responsive and effective amid changing trends in drug use.

One promising strategy for improving OST accessibility and efficiency is the implementation of low-threshold units. These units have shown positive outcomes in enhancing patient engagement and treatment retention. By lowering barriers to entry through reduced eligibility criteria and offering immediate access to treatment, low-threshold units increase efficiency and help meet the demand for OST services, particularly among underserved populations [52].

## 5. Limitations

The dataset was provided by the Organization Against Drugs (OKANA), the largest public organization focused on addiction prevention and treatment in Greece, and covered the years 2019 to 2022. Data were collected from nearly all OST treatment units operating in Greece, though four units had to be excluded due to missing data. These missing data may have affected the frontier and led to bias; moreover, the robustness and reliability of the analysis could also have been impacted [53]. While this may have slightly affected the generalizability of the findings to all OST units in Greece, the selected dataset remained representative. Furthermore, while the study relied on data provided by OKANA, no specific evidence suggested systematic bias in the reporting process. A limitation of the data was the absence of quality-focused measures beyond patient numbers, such as treatment retention or patient and staff perspectives. This highlights the need for improved monitoring to collect higher-quality data and for further research that incorporates these crucial variables.

Additionally, data collection was limited to 2022 as a restructuring process in 2023 led to the closure and merging of some treatment units [1]. This limitation highlights the need for future research to incorporate post-restructuring data to better understand how structural changes impact the efficiency and productivity of OST units. Despite these constraints, this study provided valuable insights into the operational dynamics of OST units from 2019 to 2022, laying the groundwork for further research.

## 6. Conclusions

This paper has employed the non-parametric MPI DEA method to assess changes in productivity, efficiency, and technology among OST units in Greece from 2019 to 2022. The results have provided valuable insights into the performance and contributions of each OST unit in delivering therapy, psychosocial support, and primary healthcare services to individuals facing addiction. This research aims to improve decision-making and policy development regarding resource allocation to OST units and the outcomes they achieve. It highlights the need for interventions to address new challenges and advance OST treatment in Greece. The goal is to enhance services and better meet the needs of individuals struggling with addiction.

The study’s findings offer a deeper understanding of the technical efficiency and productivity levels achieved by OST units during the examined period. The analysis showed a decrease in productivity during the first period, followed by an increase in productivity levels during the next two periods, mainly driven by technological changes. External factors such as COVID-19, new drug trends, and responses like take-home therapy and the supply of generic buprenorphine as a treatment substance influenced the analysis, providing valuable insights.

Future research should focus on comparing different treatment approaches, particularly the operational efficiency of OST units in the post-pandemic context. Further studies are also needed to explore the impact of rising synthetic opioid and stimulant use on OST treatment effectiveness. The rise in stimulant and synthetic opioid use presents new challenges for treatment programs, suggesting the need for complementary approaches alongside existing opioid substitution therapies [2,8]. Research that focuses on patient retention and adopts a more patient-centered approach—prioritizing the quality of care over operational efficiency—would be valuable. Moreover, complementing quantitative findings with qualitative data through interviews with staff and clients of OST units could provide a more holistic evaluation of the treatment experience. This would offer a deeper understanding of the challenges and successes within OST programs.

In conclusion, while the implementation of OST in Greece has made significant progress, ongoing policy adjustments and operational improvements are necessary to meet the evolving demands of addiction treatment. By enhancing data-driven decision-making, lowering treatment thresholds, and expanding access, the efficiency and effectiveness of OST programs can be improved. Additionally, addressing the needs of individuals using synthetic opioids and stimulants should be a priority in future research and treatment development. This will ensure that OST remains a comprehensive and sustainable solution for individuals seeking treatment for addiction in the future.

## Figures and Tables

**Table 1:** Descriptive statistics of inputs and outputs for the years 2019, 2020, 2021, and 2022.

Variables	Statistics	2019	2020	2021	2022
Number of Patients (monthly average)	Mean	138.796	134.611	133.981	127.574
	St. Dev.	71.035	71.213	70.258	66.604
	Min	34	30	33	30
	Max	342	322	313	312
Exams for Parallel Drug Use	Mean	5033.333	4048.055	5157.203	4440.203
	St. Dev.	2423.231	2308.128	3137.907	2646.900
	Min	15	276	136	182
	Max	10,393	12,913	18,335	12,220
Psychosocial Support Sessions	Mean	3211.166	2125.944	2328.555	3074.962
	St. Dev.	2149.564	1385.723	1994.245	3034.302
	Min	590	491	346	308
	Max	8520	7051	9728	17,883
Total Staff	Mean	8.851	10.259	9.962	9.5
	St. Dev.	3.935	4.084	4.220	4.364
	Min	1	3	2	2
	Max	21	22	22	23
Payroll	Mean	197,386.306	210,642.243	212,365.632	210,583.136
	St. Dev.	119,920.836	117,467.833	114,525.735	114,597.159
	Min	19,450.6	28,956	26,543	28,148.9
	Max	552,969.59	564,191.56	576,690.62	595,531.12
Operating Costs	Mean	31,829.728	49,463.625	46,670.727	42,703.714
	St. Dev.	16,472.947	25,931.021	24,947.269	24,210.411
	Min	11,496.46	10,908.69	11,608.04	5677.18
	Max	89,750.01	135,110.77	129,696.47	123,675.11
Medicine Costs (Normalized Costs)	Mean	1.020405927	1.018518519	1.018518519	1.018518519
	St. Dev.	0.439889694	0.414488071	0.420808803	0.380470204
	Min	0.101920058	0.114757665	0.153171942	0.145834726
	Max	1.890265396	1.861751428	2.381074881	1.921010149
Valid N (listwise)	54				

**Table 2:** Summary scores and number of OST treatment units that progressed, regressed, or remained constant from year to year.

Mean	2019–2020	2020–2021	2021–2022	Total (2019–2022)
Effch	1.026	0.908	0.953	0.961
Techch	0.649	1.209	1.118	0.958
Pech	1.000	0.952	0.980	0.977
Sech	1.026	0.954	0.972	0.984
Tfpch	0.666	1.099	1.065	0.920
DMUs that progressed (tfpch > 1)	4 (7.4%)	39 (72.3%)	36 (66.7%)	11 (20.4%)
DMUs that regressed (tfpch < 1)	50 (92.6%)	15 (27.7%)	18 (33.3%)	43 (79.6%)
DMUs remained constant (tfpch = 1)	0	0	0	0

**Table 3:** Summary results: number of OST treatment units that progressed, regressed, or remained constant.

PERIOD (Comparing Years)	2019–2020	2020–2021	2021–2022	Total (2019–2022)
Change into Effch				
Prog. (effch > 1)	23 (42.6%)	9 (16.7%)	19 (35.2%)	15 (27.8%)
Reg. (effch < 1)	21 (38.9%)	33 (61.1%)	24 (44.4%)	29 (53.7%)
Constant (effch = 1)	10 (18.5%)	12 (22.2%)	11 (20.4%)	10 (18.5%)
Change into Techch				
Prog. (techch > 1)	1 (1.9%)	50 (92.6%)	38 (70.4%)	14 (26%)
Reg. (techch < 1)	53 (98.1%)	4 (7.4%)	16 (29.6%)	40 (74%)
Constant (techch = 1)	0	0	0	0
Change into Tfpch				
Prog. (tfpch > 1)	4 (7.4%)	39 (72.2%)	36 (66.7%)	11 (20.4%)
Reg. (tfpch < 1)	50 (92.6%)	15 (27.8%)	18 (33.3%)	43 (79.6%)
Constant (tfpch = 1)	0	0	0	0

## Data Availability

The data that support the findings of this study are available from the Organization Against Drugs (OKANA), but restrictions apply to the availability of these data, which were used under license for the current study and so are not publicly available. Data are however available from the authors upon reasonable request and with the permission of the Organization Against Drugs (OKANA).

## Abbreviations

The following abbreviations have been used in this manuscript:

OKANA	Organization Against Drugs
EMCDDA	Europeans Monitor Center for Drugs and Drug Addictions
EUDA	European Union Drugs Agency
OST	Opioid Substitution Treatment
DEA	Data Envelopment Analysis
DMU	Decision-Making Units
CRS	Constant Return to Scale
VRS	Variable Return to Scale
MPI	Malmquist Productivity Index
